# Advances in subcutaneous injections: PRECISE II: a study of safety and subject preference for an innovative needle-free injection system

**DOI:** 10.1080/10717544.2021.1976309

**Published:** 2021-09-20

**Authors:** E. Lynne Kelley, Richard H. Smith, Gillian Corcoran, Sandra Nygren, Mary V. Jacoski, Andrea Fernandes

**Affiliations:** Portal Instruments, Cambridge, MA, USA

**Keywords:** Needle-free injector, pain, preference, subcutaneous injection, self-injection

## Abstract

Needle-free injection is a desirable goal for many reasons, including reducing pain, anxiety, and eliminating safety risks associated with needle-stick injuries. However, development of a safe, reliable needle-free device optimized for at-home use has been met with many challenges. Portal Instruments Inc. has been developing needle-free medication delivery using a well-designed hand-held device, PRIME, that is safe, intuitive to use, and utilizes advanced electronic control of a focused, high velocity, pressurized liquid injection stream. The PRECISE II human study demonstrated that the PRIME needle-free injection system was safe, well tolerated, and strongly preferred by participants for self-injections over a standard needle and syringe. In addition, the study was able to be completed early for superiority following the success of the pre-defined interim analysis.

## Introduction

Advances in medication therapies have improved the lives of countless patients facing the challenge of chronic diseases. Adherence to prescribed therapy is key to achieving desired clinical outcomes in all adverse health conditions. Patient compliance is particularly critical when treatment requires regular, intermittent injections of medications. However, suboptimal treatment adherence and discontinuation of therapy due to needle phobia or injection pain remains a major challenge for clinicians and their patients treated with self-injectable medications (Cox & Stone 2006; Deacon & Abramowitz 2006; Devonshire et al. 2011; Orenius et al. 2018). Injection anxiety due to pain and needle phobia is very common among patients with relapsing-remitting multiple sclerosis who self-inject, which can pose difficulties for medication compliance where most of the currently marketed effective medications are self-injectables (Cox & Stone 2006). An observational, multi-national study of 2,648 patients using self-injection therapy for treatment of relapsing-remitting multiple sclerosis revealed an overall adherence rate of 75%; however, 30% of non-adherent patients cited adverse skin reactions, injection-site pain or anxiety about injections as their primary reason for non-adherence (Devonshire et al. 2011). Similar associations between non-adherence and injection discomfort and anxiety have also been demonstrated among pediatric and adult patients receiving growth hormone therapy (Rosenfeld & Bakker 2008). In addition to injection anxiety and discomfort, the use of needle and syringe-based devices and even autoinjectors for medication self-injection can also be challenging in chronic medical conditions with associated dexterity-related struggles such as in rheumatoid arthritis (Schiff et al. 2016; van den Bemt et al. 2019).

Needle phobia is a prevalent and yet under recognized health issue. Fear of needles is common in both children (∼33–63%) and adults (∼14–38%) (Orenius et al. 2018) and can contribute to poor adherence among patients as well as negative experiences for caregivers, clinicians and health professionals (Deacon & Abramowitz 2006; Orenius et al. 2018). A study by Taddio and colleagues estimated that as many as 2 out of 3 people are afraid of needles (Taddio et al. 2012). The relatively high prevalence of needle phobia also raises significant health concerns in light of the COVID-19 pandemic, as fear of needles can lead to vaccine avoidance (Taddio et al. 2015; Love & Love 2021). Finally, the use of needle and syringe devices have an added risk of needle-stick injuries and require special needle disposal.

Autoinjectors simplify at-home self-injection of medications, but although hidden, they still contain a needle. Additionally, autoinjectors require the user to press and hold the device against the skin for up to 15 seconds, and larger volumes can require up to 30 seconds (Schneider et al. 2020). Over the past several years, technologies that use needle-free injection methods have been developed to address the challenges associated with injection pain and needle anxiety. A spring-loaded reusable jet-injector has demonstrated positive data for vaccine delivery, although use has been limited to trained healthcare professionals (de Menezes Martins et al. 2015; Basu et al. 2021). Among the newest devices is the Portal PRIME needle-free jet injection system (Portal Instruments, Inc., Cambridge, MA). Where previousneedle-free injection systems relied on mechanical spring-based or gas-based approaches to achieve the high pressure required to eject at a velocity necessary to pierce the skin, the lack of real-time control limited their application for medications that require larger volumes and those with higher viscosity (Taberner et al. 2012). The Portal PRIME needle-free injector delivers a narrow stream of medication through the skin in less than half a second using technology that controls and modifies the fluid velocity in real time by employing a feedback control loop connected to an electro-mechanical actuator that generates the force needed to inject the fluid.

Exploratory studies of earlier versions of the Portal technology showed a safety profile similar to injection with a 27-gauge needle and syringe (Kojic et al. 2017). Although the pain associated with needle-free injection was comparable to needle and syringe, overall user preference strongly favored the needle-free method (Kojic et al. 2017). In this report, we present findings from the first-in-human, randomized, crossover study (PRECISE II) that investigated the safety profile and patient preference associated with the Portal PRIME device, as well as assessing the ability of subjects to self-inject using this needle-free device.

## Materials and methods

**Figure 1.**
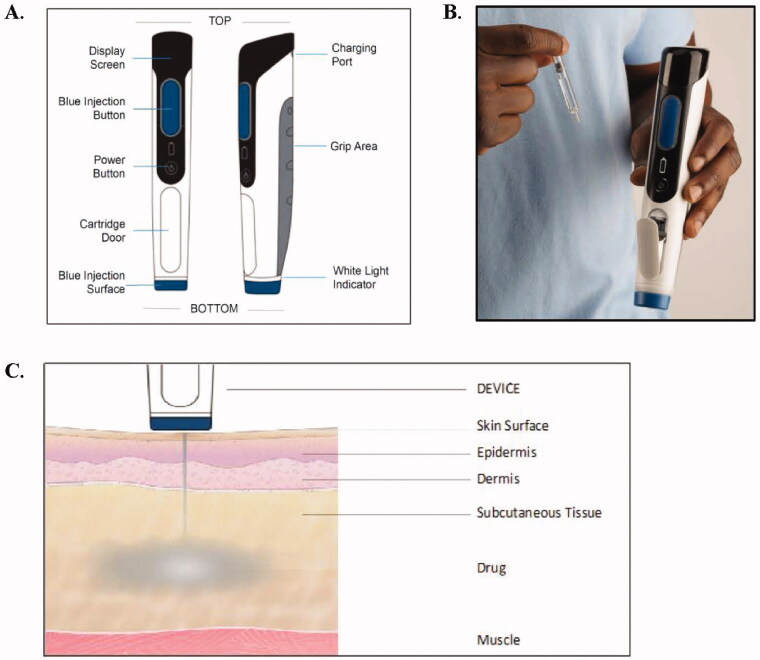
PRIME Device (A), the PRIME Device and Disposable Cartridge (B), and the Injection Process (C).

The Portal PRIME Needle-free Injection System consists of a reusable computer-driven handheld injector and a single-use, sterile needle-free cartridge that can be filled with liquid medication using standard production techniques (Figure 1(A,B)). PRIME employs a method of force generation using a linear electro-magnetic motor that propels liquid through a small orifice in the cartridge (<200 µm in diameter) to enable the delivery of a biologic or therapeutic drug rapidly and directly through the skin using a pressurized liquid stream (Figure 1(C)). PRIME delivers liquids over a wide range of viscosities by creating a focused, high velocity fluid jet which penetrates the skin and rapidly injects the medication into the subcutaneous tissue. A 1.0 mL injection, for example, is completed in less than 0.34 seconds. During the injection period, the device is constantly monitoring and adjusting the velocity and volume of injection to ensure a precise and complete injection (Taberner et al. 2012).

Subcutaneous injections with PRIME are achieved through a two-phase injection profile: 1) Pierce Phase and 2) Follow-Through/Fill Phase. In the Pierce Phase, a fast fluid jet pierces the skin to a desired tissue depth (e.g. subcutaneous space). In the Follow-Through/Fill Phase, the jet velocity is decreased to ensure that the drug does not penetrate deeper and a ‘filling’ at the desired depth (subcutaneous) occurs. During the injection period, the device is constantly monitoring and adjusting the velocity and volume of injection to ensure a precise and complete injection. For a 0.68 mL injection, the Pierce Phase is about 50 ms and the Follow-Through/Fill Phase is approximately 150 ms. Therefore, total injection time for a 0.68 mL dose is typically 200 ms.

### Study design

This prospective, single-center, crossover study (PRECISE II) assessed the safety and participant preferences of two injection techniques in a cohort of healthy adult volunteers. Participants self-injected 0.68 mL of sterile saline (NaCl 0.9%) subcutaneously using a prefilled syringe (PFS) with 27-gauge needle and the Portal needle-free injection system (PRIME). Each participant performed three injections at one anatomic location separated by a minimum distance of 50 mm and in time by 10 minutes.

The study was conducted under the requirements outlined in current Good Clinical Practices. An independent Institutional Review Board (IntegReview IRB, Austin, TX) reviewed and approved the study protocol, informed consent form, recruitment materials and all amendments to these documents. All participants provided written informed consent to participate in the study after having been informed about the nature and purpose of the study, participation/termination conditions, and risks and benefits of treatment. Consent was obtained prior to the performance of any study procedures.

### Subjects and randomization

Forty-two healthy adult subjects were enrolled in the study, having met the following criteria: ≥18 years of age; willing to sign informed consent, willing perform self-injections, willingness to undergo digital photography limited to the injection sites and comply with follow up procedures, including telephone and/or email communication; and able to read and speak English. Exclusion criteria included: known pregnancy or breastfeeding; presentation with rash, pigmentation, lesions, scars, tattoos or other abnormal skin conditions in the abdominal or upper thigh area; current or recent (previous 12 weeks) treatment with anti-coagulant/blood thinner medications; any known history or active recurrent bacterial, viral, fungal, mycobacterial or other infections; recent (within 14 days) abdominal or upper thigh injections; upper arm motor limitations that would prevent holding and applying the injection device against the skin of the abdomen or thigh; prior participation in a Portal Instruments device injection study.

Participants were randomized for injection site (abdomen vs. thigh; left vs. right side) and order of injection method for the first two injections to minimize any potential for bias using a computer-generated randomization method with block sizes of 4 and 6. For the third and final injection, participants were asked to choose which of the two methods of injection they would prefer to repeat in the same anatomical region to which they were randomized. Each participant administered a total of 3 injections of up to 1 mL each. The sequence that participants were randomly assigned to informed which injection method the participant handled first. In Sequence 1, participants were introduced to the PFS first, followed by PRIME. In Sequence 2, participants were introduced to PRIME first, followed by the PFS. Participants were then asked their preference for injection method for the third injection and performed that injection in the same anatomical area. Randomization was performed by an individual who was not a part of the clinical study team and study coordinators remained blinded until after randomization on Day 1 of each participant entering the study. The active portion of the study was not blinded.

Preference was assessed in four areas: the preferred injection method, which injection method was easier to use, which method was preferred in the setting of a need for chronic self-injections of medication, and which methods would lead to improved compliance. Preference was assessed with a 5-point Likert Scale by addressing statements with the following possible response categories: Strongly Prefer/Much Better for either method, Somewhat Prefer/Somewhat Better for either method and No Preference/Same with Either Injector. The number of responses in each category were tabulated for each statement.

Safety was assessed objectively through visual examination performed by a licensed healthcare professional and by the participants through participant-reported outcomes. Injection sites were assessed at time 0 (immediately, within 2 min), 5 min and 10 min after each injection for injection site reactions (tenderness, redness, swelling) according to the toxicity scale provided by FDA guidance (FDA 2007). Each participant also rated the pain sensation experienced at each injection site at the same timepoints using a standard 100 mm visual analog scale (VAS). All adverse events were documented during the study visit as well as during the follow-up procedure that occurred 24–48 hours following the study visit.

### Sample size

This study was designed to show that subjects preferred PRIME over the PFS more frequently than random chance alone. With two methodologies, there was a 50% chance of preferring one approach over the other by chance alone; however, a more conservative probability of 55% was used as the minimum threshold. Based on previous studies, it was expected that roughly 70% of the participants would prefer PRIME over the PFS. A total sample size of 84 participants evaluating the two injection methods would yield 80% power to declare 70% statistically larger than 55% when using a two-stage sequential analysis using chi-square tests with an overall one-sided alpha = 0.025 of statistical significance. An interim analysis was prespecified following enrollment and completion of the first 42 participants. This report describes the results of this pre-planned interim analysis.

### Statistical analysis

The data, regardless of injection order or location, were pooled and analyzed together. The interim analysis was planned following enrollment and completion of 42 participants and was performed by a statistician independent from the protocol statistician. The interim look provided the opportunity to review the safety of PRIME and consider terminating the study in the event of a safety concern or if the superiority threshold for statistical significance of the interim analysis was achieved.

The primary objective of this study was to show that participants prefer PRIME over the PFS more frequently than random chance alone. Each participant assessed their preference for using PRIME or using the PFS using a 5-point Likert Scale. The dichotomous variable PRIME Preferred was classified as ‘YES’ if the participant marked on the Likert scale ‘Strongly Prefer the Needle-Free Injector’ or ‘Somewhat Prefer the Needle-Free Injector’ as to their preference to using the PRIME over using a PFS; otherwise, PRIME Preferred was classified ‘NO’.

The one-sided chi-squared test was used to evaluate the hypothesis H0: πPRIME ≤0.55 versus H1: πPRIME > 0.55 using a two-stage sequential analysis using chi-square tests with an overall one-sided alpha = 0.025 of statistical significance. The 95% Confidence Interval for the percentage of participants preferring the PRIME is provided to aid in the interpretation of the results. Should the uncorrected one-sided p-value for the primary efficacy endpoint be less than or equal to 0.016, the null hypothesis may be rejected, the PRIME Preference Rate would be declared statistically greater than 55%, and enrollment stopped. The independent statistician made a study stop/continuation recommendation based on the Per-Protocol analysis cohort. If the study were to continue, the uncorrected one-sided p-value for the primary efficacy endpoint for the fully enrolled study should be less than or equal to 0.014 for the null-hypothesis to be rejected and the primary endpoint declared a success.

Dichotomous (e.g. PRIME Preferred) and ordinal (e.g. Injector Preference by Likert Scale) data were tabulated by category. The mean, standard deviation, median, maximum, and minimum were tabulated for continuous data (e.g. age). The significance level was two-sided 0.05 for all statistical tests.

## Results

A total of 42 healthy adult human volunteers with an average age of 39.1 years (range 19–87) were consented and participated in this interim analysis of the study. Randomization resulted in 20 abdominal locations and 22 thigh locations. All participants completed all injections and questionnaires. Of the 42 participants, 24 were female (57%, average age 35.0 years) and 18 were male (43%, average age 44.6 years). Average height among participants was 66.9 inches (range 58–75 inches), and average weight was 170.9 lb., (range 110–275 lb.). Caucasians accounted for 81.0% of participants (*n* = 34), while 9.5% were black/African American (*n* = 4), 2 were Asian/Pacific Islander, and one each of Indian and Middle Eastern race.

No participants were removed from the study, but one participant was lost to follow up for collection of a final resolution of a mild adverse event. There was one protocol violation, as one subject only reported preference for the third injection, but did not complete the injection itself, therefor all secondary pain and tolerability data were missing for that final injection. Therefor the 42 participants completed a total of 75 injections using the PRIME device, and 50 injections by the needle and syringe.

There were no deaths or serious adverse events during the study, and no participant discontinued the study due to an adverse event. There were seven adverse events in five participants, and were limited to itchiness, tenderness, ecchymosis and swelling at the injection site. All were mild and resolved without any medical intervention, except for one instance of ecchymosis that occurred in a participant who was subsequently lost to follow up. However, the ecchymosis was reported as resolving over the 12 days prior to the subject terminating follow-up contact. See Table 1.

**Table 1.** Adverse events.

Subject	1st injection type	Event injection type (location)	Description	Severity	Relationship	Resolution
Subject-018	PRIME	PRIME (abdomen)	Intermittent itchiness left side of abdomen at first PRIME injection site	Mild	Definitely Related	Resolved, no sequelae
Subject-020	PFS	PRIME (abdomen)	Ecchymosis first PRIME injection	Mild	Definitely Related	Resolving at time of lost to follow up^a^
Subject-042	PFS	PRIME (abdomen)	Tenderness at first and second PRIME injection sites	Mild	Definitely Related	Resolved, no sequelae
Subject-057	PFS	PFS (thigh)	Ecchymosis at PFS injection site	Mild	Definitely Related	Resolved, no sequelae
Subject-059	PFS	PRIME (abdomen)	Ecchymosis first and second PRIME injection sites	Mild	Definitely Related	Resolved, no sequelae

^a^Subject 20 randomized to the abdomen injection location and the PFS for the first injection. He chose the PRIME injection for his third injection method. Subject strongly preferred the PRIME device when answering the preference questions. Images taken of the injection sites at time of study showed minimal erythema. He reported ecchymosis at the first of the PRIME injection sites at the 24–48 hour follow up. He reported resolving ecchymosis over the next 12 days but stopped responding to telephone calls or emails to allow for final resolution.

### VAS pain scores

**Figure 2.**
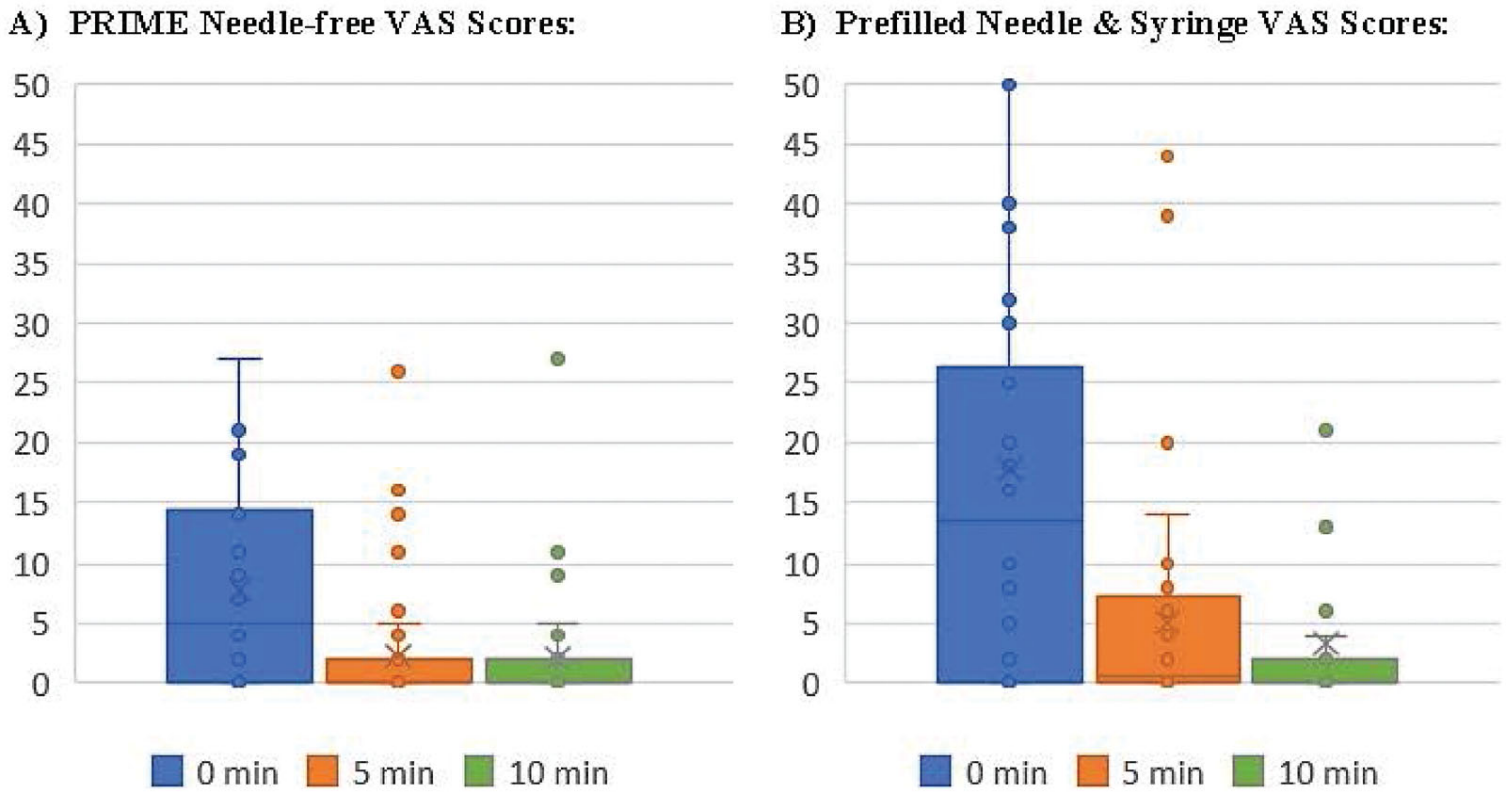
Distribution of VAS Pain Scores (in mm) at 0 minutes, 5 minutes and 10 minutes for Assigned (A) PRIME Needle-free and (B) Prefilled Needle & Syringe Injections.

The average pain scores reported by the 42 participants were significantly lower with the PRIME injection system compared with the PFS immediately post injection through 5 minutes when evaluating the assigned injections (Figure 2, Table 2), which excludes the third ‘by choice’ injections. The average VAS score immediately post injection was 7.8 mm (range 0–27) for PRIME injections, compared to 17.7 mm (0–100) for PFS injections. VAS scores decreased over time post injection for both methods with statistically significant differences at the two earlier time points, and no difference in pain at the 10-minute evaluation.

**Table 2.** Average VAS pain scores for PRIME and PFS study injections.

0 Minutes			5 Minutes			10 Minutes		
PRIME mean (SD)	PFS mean (SD)	*p* Value	PRIME mean (SD)	PFS mean (SD)	*p* Value	PRIME mean (SD)	PFS mean (SD)	*p* Value
7.8 (8.2)	17.7 (20.0)	.003	2.3 (5.3)	5.1 (9.5)	.032	2.1 (4.8)	3.3 (9.3)	.249

Data are presented as mean (standard deviation), all in millimeters. The mean and standard deviation of 42 subjects’ VAS scores are presented for each injection method at each timepoint. P-value calculated using a paired *T*-test of two-tailed probability.

Subjects were also assessed regarding pain or tenderness and any observations at the 5- and 10-minute post injection period using the standardized FDA assessment for vaccines. Tenderness was reported infrequently in both groups and was short-lived. There were a small number of participants in both injection types that reported other observations, which were limited to itching or burning sensations and drops of blood at injection sites. Importantly there were no observations of any events greater than mild or Grade 1 of persistent induration/swelling or erythema/redness following either injection method, although the number of injection sites with minor measurable reactions were greater in the needle-free device compared to the prefilled needle and syringe in both categories (Table 3).

**Table 3.** Additional safety assessments^a^.

Local reaction to saline injection	Any observations		Mild (Grade 1)		Moderate (Grade 2)		Severe (Grade 3)		Potentially Life Threatening (Grade 4)	
Injection Method:	PRIME	PFS	PRIME	PFS	PRIME	PFS	PRIME	PFS	PRIME	PFS
Tenderness at 5 minutes, events	2/75	3/50	2	3						
Tenderness at 10 minutes, events	1/75	0/49	1	0						
Other Observations at 5 minutes, events	0/75	2/50	0	2						
Other Observations at 10 minutes, events	1/75	1/49	1	1	No observations of any events greater than mild or Grade 1 for either injection method					
Erythema/Redness at 5 minutes	23/75	1/50	0	0						
Ave diameter (cm), those with event	0.72	1.0	0	0						
Erythema/Redness at 10 minutes, events	21/75	0/49	0	0						
Ave diameter, (cm) those with event	0.65	0	0	0						
Induration/Swelling at 5 minutes	49/75	1/50	0	0						
Ave diameter (cm), those with event	0.54	0.30	0	0						
Induration/Swelling at 10 minutes, events	35/75	2/49	1/75^b^	1/49^c^						
Ave diameter (cm), those with event	0.42	0.20	4.0	0.2						

^a^Data were available for *n* = 41 participants, and *n* = 40 patients for the 10-minute measures.

^b^Induration/swelling measures for PRIME injections in one subject were reported as 4.0 cm, however the photos of those injection site did not appear to be that large.

^c^Induration/swelling was reported as Grade 1 for the PFS injection at 10 minutes in one subject, however the measured diameter was only reported as 0.2 cm, which does not meet the criteria for Grade 1.

### Subject preference

**Figure 3.**
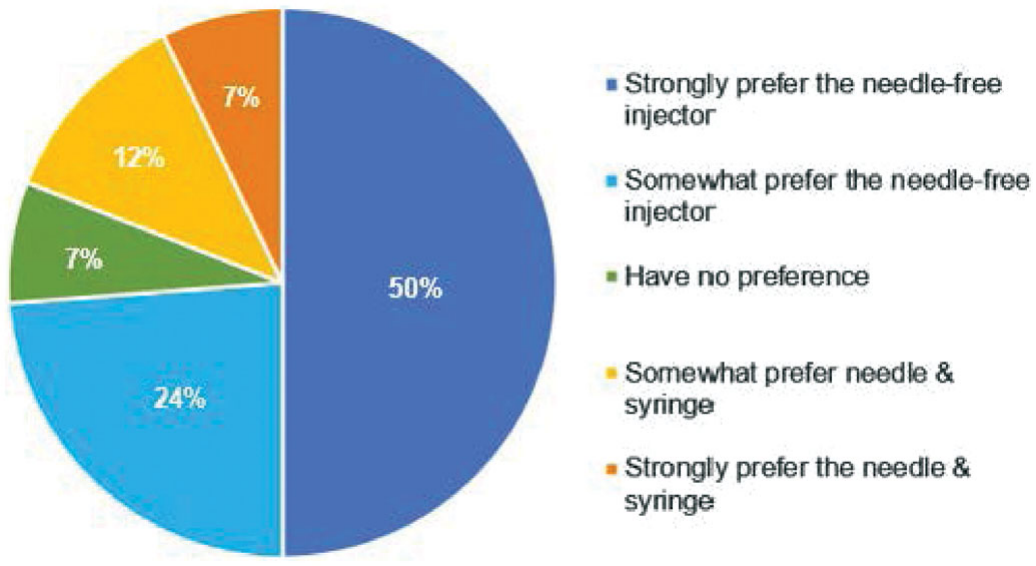
Preference. Response to the question: ‘When comparing the Needle-free Injector to the Prefilled Needle & Syringe, I:’.

At the completion of the first two self-injections, participants were provided a questionnaire to determine whether there was a preference between the two different injection methods. Figure 3 reveals that the majority (76%) of participants either strongly prefer (52%) or somewhat prefer (24%) the PRIME device over the PFS. This study was stopped following completion of this pre-defined interim analysis, as the PRIME preference rate was significantly greater than 55%, with an uncorrected one-sided *p*-value <.016.

Preference results were similar when evaluated by location, with 65% of those randomized to the abdomen and 82% of those randomized to the thigh preferring PRIME over the PFS method. When it was time to choose the method of injection for the third, voluntary self-injection, 33 participants (79%) selected the PRIME injection method, and 9 participants (21%) selected the PFS method. Again, this preference was similar when stratified by location of the injections: 17/22 (77%) of participants randomized to thigh injections selected PRIME as their preferred method, while 16/20 (80%) of those randomized to the abdomen selected PRIME as their preferred method.

**Figure 4.**
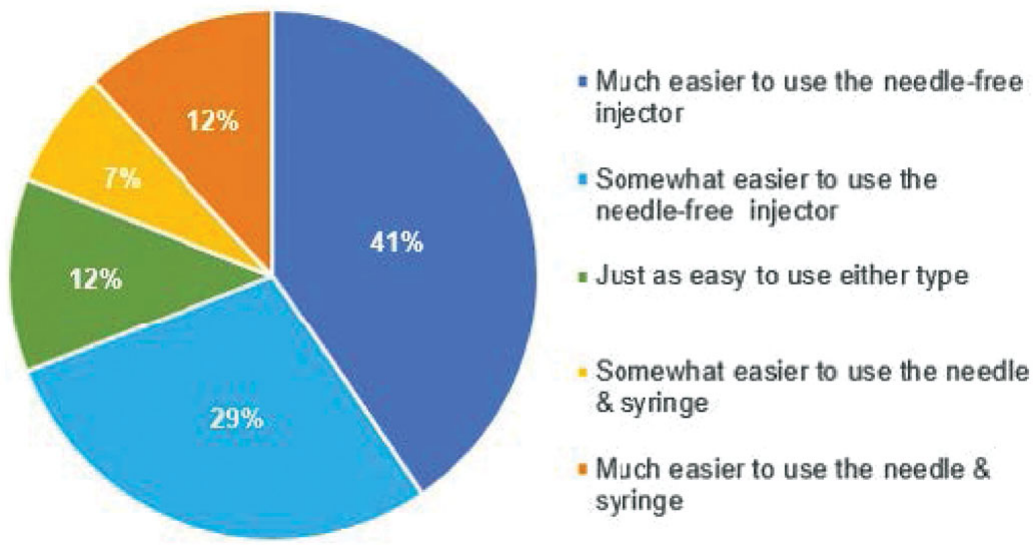
Ease of Use. Response to the question: ‘When comparing the Needle-free Injector to the Prefilled Needle & Syringe, it is:’.

When asked to compare the ease of use of the two injection types, the majority (70%) of subjects indicated that the PRIME injection system was either *much easier* (41%) or *somewhat easier* (29%) than injections using the PFS (Figure 4).

**Figure 5.**
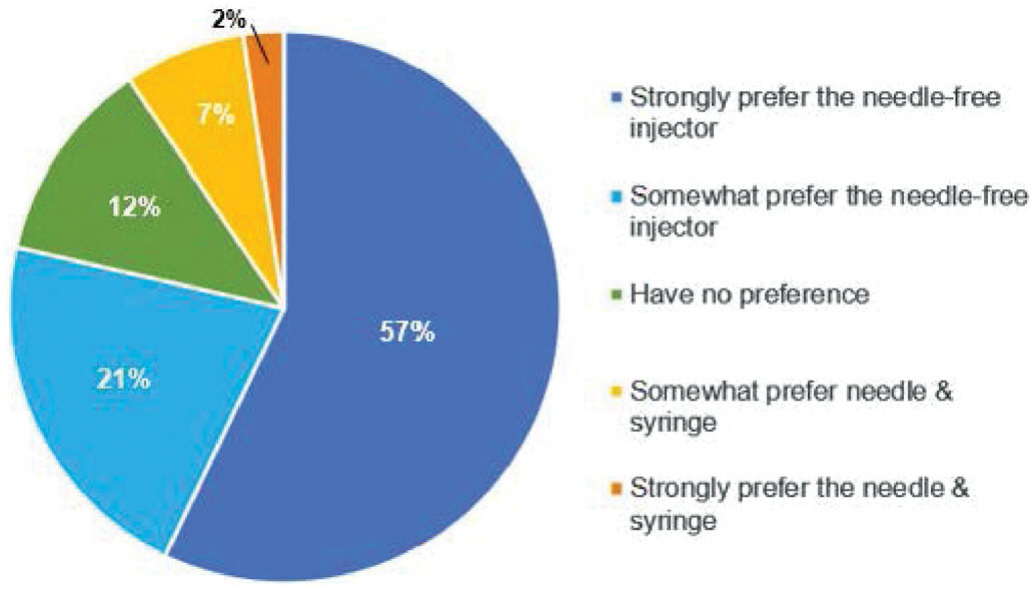
Quality of Life. Response to the questions: ‘If you had to inject yourself on a weekly basis because of a chronic illness, which device would you want your physician to prescribe?’.

When subjects were asked their preference of injection method in a scenario where they were diagnosed with a chronic disease requiring weekly injections, the majority (78%) indicated that they would *strongly prefer* (57%) or *somewhat prefer* (21%) the PRIME needle-free injection device over the needle and syringe (Figure 5).

**Figure 6.**
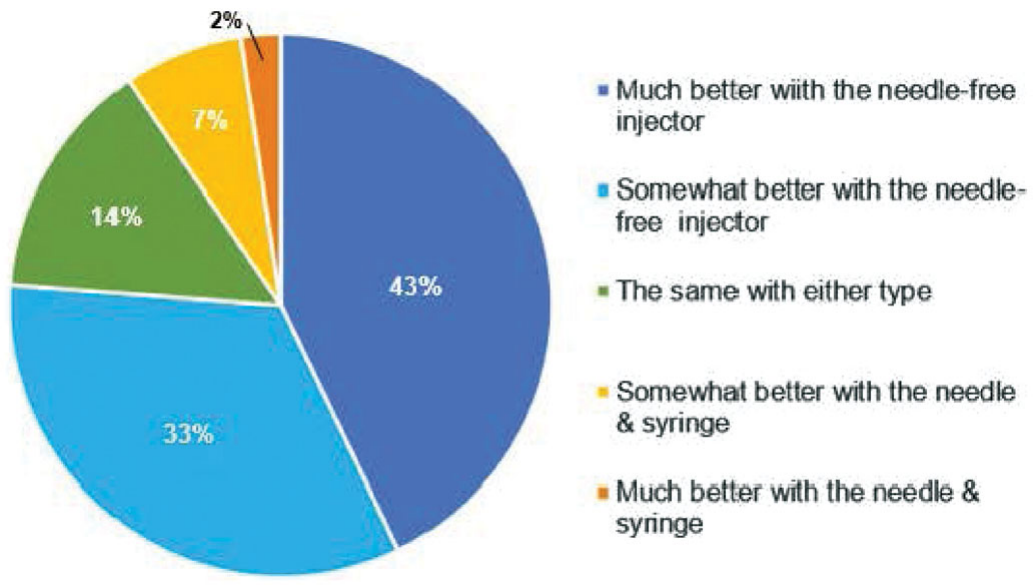
Medication Adherence. Response to the question: ‘I think the chances that I would take my medication would be:’.

The final preference question assessed a potential impact on medication compliance. The majority (76%) of subjects indicated that the needle-free injector would have a positive impact with 43% ranking a much better chance and 33% ranking a somewhat better chance when compared to the standard needle and syringe injections (Figure 6).

## Discussion

This clinical study demonstrated that self-injections into the abdomen and upper lateral thigh using the PRIME needle-free injection system were safe and well-tolerated. Additionally, subjects strongly preferred the use of the PRIME system for self-injections versus a standard needle and syringe. In addition to the strong preference of PRIME over the standard needle and syringe injection method, the PRIME system was also preferred by subjects when queried regarding ease of use, having to self-inject weekly to manage chronic health conditions, and in the potential impact on improving injectable medication compliance. The study was terminated following the pre-defined interim analysis of the first 42 subjects enrolled, as the statistical success criteria for the superiority of interim analysis of subjects preferring the PRIME device were met. The average pain scores reported using the VAS scale were also significantly lower for PRIME compared to the prefilled needle and syringe (PFS) immediately post injection through five minutes post injection. While the average pain score reported immediately post injection for the PRIME injections (7.8 mm) was significantly lower than the PFS injections (17.7 mm), the result for the PFS was similar to that reported in other studies (Berteau et al. 2015; Heise et al. 2014). Importantly, there were no serious adverse events or significant reports of tenderness or injection site reactions with either method. The results of this study will inform future studies, as the PRIME device has the potential to be transformative in drug delivery.

The evolution of the Portal Instruments devices has progressed from a large table-top design that required a healthcare professional to assist in the injection (Kojic et al. 2017) to the current ergonomic PRIME device that allows for self-injection, fits in the hand, is rechargeable and has clear visual indicators to ensure all steps are followed for the injection to be successful. The ability to use the PRIME device in two different anatomic locations provides additional flexibility for patients to rotate injection sites, further improving the safety profile. The ability to inject in the upper lateral thigh is particularly valuable given its light distribution of cutaneous nerve fibers and lack of important neurovascular structures. Also, unlike autoinjectors which are single use devices, the PRIME device is reusable, requiring only disposable pre-filled cartridges for drug delivery. The lack of any needles allows for improved safety for caregivers and patients in that there is no risk of needle-stick injuries, and no sharps disposal requirements of the cartridge following completion of injections.

Importantly, participants who had never been exposed to the PRIME needle-free device were able to perform all the key steps for a successful injection with minimal instruction and the ability to read the proposed package insert. They were able to perform the self-injection with either no assistance or minor suggestions for correct practice. These data are important in validating the ease of use of the PRIME system, as there have been many features built into this handheld, lightweight device to ensure the proper loading of the cartridge, positioning and pressure on the skin for safe and effective injections, and clear signals for informing when an injection is complete.

## Conclusion

In summary, the PRIME needle-free injection system has been demonstrated in this safety study for upper lateral thigh and abdominal injections to be a well-tolerated method that was strongly preferred by subjects when compared to a standard needle and syringe injection. Participants were able to use the device effectively with minimal instruction. The advancement of needle-free injection technology of the PRIME device has potential positive implications especially for patients who require frequent subcutaneous injections of medications for a variety of conditions. Also, given the urgent need for successful implementation of a national COVID-19 vaccination program, more widespread utilization of needle-free injection options is needed to the encourage individuals to receive the vaccination. Further studies are needed to continue to validate the device as an alternative to both needle and syringe and autoinjectors, as well as to confirm effective drug delivery and tissue distribution as comparable to that injected by standard needle and syringe.

## References

[CIT0001] Basu I, Agarwal M, Shah V, et al. (2021). Immunogenicity and safety of two quadrivalent influenza vaccines in healthy adult and elderly participants in India - A phase III, active-controlled, randomized clinical study. Hum Vaccin Immunother 1–10. doi:10.1080/21645515.2021.1885278. PMC8920161 33957854

[CIT0002] Berteau C, Filipe-Santos O, Wang T, et al. (2015). Evaluation of the impact of viscosity, injection volume, and injection flow rate on subcutaneous injection tolerance. Med Devices 8:473–84. 10.2147/MDER.S91019 PMC4646585 26635489

[CIT0003] Cox D, Stone J. (2006). Managing self-injection difficulties in patients with relapsing-remitting multiple sclerosis. J Neurosci Nurs 38:167–71. 16817668 10.1097/01376517-200606000-00005

[CIT0004] de Menezes Martins R, Curran B, Maia Mde L, et al. (2015). Immunogenicity and safety of measles-mumps-rubella vaccine delivered by disposable-syringe jet injector in healthy Brazilian infants: a randomized non-inferiority study. Contemp Clin Trials 41:1–8. 25476584 10.1016/j.cct.2014.11.014

[CIT0005] Deacon B, Abramowitz J. (2006). Fear of needles and vasovagal reactions among phlebotomy patients. J Anxiety Disord 20:946–60. 16460906 10.1016/j.janxdis.2006.01.004

[CIT0006] Devonshire V, Lapierre Y, Macdonell R, et al.; for the GAP Study Group. (2011). The Global Adherence Project (GAP): a multicenter observational study on adherence to disease-modifying therapies in patients with relapsing-remitting multiple sclerosis. Eur J Neurol 18:69–77. 20561039 10.1111/j.1468-1331.2010.03110.x

[CIT0007] FDA. 2007. Toxicity grading scale for healthy adult and adolescent volunteers enrolled in preventive vaccine clinical trials. CBER. 10.1016/j.vaccine.2023.07.072 37532612

[CIT0008] Heise T, Nosek L, Dellweg S, et al. (2014). Impact of injection speed and volume on perceived pain during subcutaneous injections into the abdomen and thigh: a single-centre, randomized controlled trial. Diabetes Obes Metab 16:971–6. 24720741 10.1111/dom.12304

[CIT0009] Kojic N, Goyal P, Lou CH, Corwin MJ. (2017). An innovative needle-free injection system: comparison to 1 ml standard subcutaneous injection . AAPS PharmSciTech 18:2965–70. 28462463 10.1208/s12249-017-0779-0

[CIT0010] Love AS, Love RJ. (2021). Considering needle phobia among adult patients during mass COVID-19 vaccinations. J Prim Care Community Health 12:21501327211007393. 33813931 10.1177/21501327211007393 PMC8020217

[CIT0011] Orenius T, LicPsych H, Saila K, et al. (2018). Fear of injections and needle phobia among children and adolescents: an overview of psychological, behavioral, and contextual factors. SAGE Open Nurs 4:2377960818759442. 33415191 10.1177/2377960818759442 PMC7774419

[CIT0012] Rosenfeld RG, Bakker B. (2008). Compliance and persistence in pediatric and adult patients receiving growth hormone therapy. Endocr Pract 14:143–54. 18308651 10.4158/EP.14.2.143

[CIT0013] Schiff M, Koo J, Jin E, et al. (2016). Usability and acceptability of the abatacept pre-filled autoinjector for the subcutaneous treatment of rheumatoid arthritis. Adv Ther 33:199–213. 26833303 10.1007/s12325-016-0286-9 PMC4769728

[CIT0014] Schneider A, Mueller P, Jordi C, et al. (2020). Hold the device against the skin: the impact of injection duration on user's force for handheld autoinjectors. Expert Opin Drug Deliv 17:225–36. 31835921 10.1080/17425247.2020.1704730

[CIT0015] Taberner A, Hogan NC, Hunter IW. (2012). Needle-free jet injection using real-time controlled linear Lorentz-force actuators. Med Eng Phys 34:1228–35. 22245386 10.1016/j.medengphy.2011.12.010

[CIT0016] Taddio A, McMurtry CM, Shah V, et al. (2015). Reducing pain during vaccine injections: clinical practice guideline. CMAJ 187:975–82. 26303247 10.1503/cmaj.150391 PMC4577344

[CIT0017] Taddio A, Ipp M, Thivakaran S, et al. (2012). Survey of the prevalence of immunization non-compliance due to needle fears in children and adults. Vaccine 30:4807–12. 22617633 10.1016/j.vaccine.2012.05.011

[CIT0018] van den Bemt BJF, Gettings L, Domańska B, et al. (2019). A portfolio of biologic self-injection devices in rheumatology: how patient involvement in device design can improve treatment experience. Drug Deliv 26:384–92. 30905213 10.1080/10717544.2019.1587043 PMC6442222

